# Hydrogen Peroxide Contributes to the Epithelial Cell Death Induced by the Oral Mitis Group of Streptococci

**DOI:** 10.1371/journal.pone.0088136

**Published:** 2014-01-31

**Authors:** Nobuo Okahashi, Tomoko Sumitomo, Masanobu Nakata, Atsuo Sakurai, Hirotaka Kuwata, Shigetada Kawabata

**Affiliations:** 1 Department of Oral Frontier Biology, Osaka University Graduate School of Dentistry, Suita-Osaka, Japan; 2 Department of Oral and Molecular Microbiology, Osaka University Graduate School of Dentistry, Suita-Osaka, Japan; 3 Department of Pediatric Dentistry and Oral Health Science Center hrc8, Tokyo Dental College, Chiba, Japan; 4 Department of Oral Microbiology and Immunology, Showa University School of Dentistry, Tokyo, Japan; Columbia University, United States of America

## Abstract

Members of the mitis group of streptococci are normal inhabitants of the commensal flora of the oral cavity and upper respiratory tract of humans. Some mitis group species, such as *Streptococcus oralis* and *Streptococcus sanguinis*, are primary colonizers of the human oral cavity. Recently, we found that hydrogen peroxide (H_2_O_2_) produced by *S. oralis* is cytotoxic to human macrophages, suggesting that streptococcus-derived H_2_O_2_ may act as a cytotoxin. Since epithelial cells provide a physical barrier against pathogenic microbes, we investigated their susceptibility to infection by H_2_O_2_-producing streptococci in this study. Infection by *S. oralis* and *S. sanguinis* was found to stimulate cell death of Detroit 562, Calu-3 and HeLa epithelial cell lines at a multiplicity of infection greater than 100. Catalase, an enzyme that catalyzes the decomposition of H_2_O_2_, inhibited *S. oralis* cytotoxicity, and H_2_O_2_ alone was capable of eliciting epithelial cell death. Moreover, *S. oralis* mutants lacking the *spxB* gene encoding pyruvate oxidase, which are deficient in H_2_O_2_ production, exhibited reduced cytotoxicity toward Detroit 562 epithelial cells. In addition, enzyme-linked immunosorbent assays revealed that both *S. oralis* and H_2_O_2_ induced interleukin-6 production in Detroit 562 epithelial cells. These results suggest that streptococcal H_2_O_2_ is cytotoxic to epithelial cells, and promotes bacterial evasion of the host defense systems in the oral cavity and upper respiratory tracts.

## Introduction

Members of the mitis group of streptococci are major inhabitants of the commensal flora of the oral cavity and upper respiratory tract of humans [1], [2]. The mitis group includes *Streptococcus pneumoniae*, *Streptococcus mitis*, *Streptococcus oralis*, *Streptococcus sanguinis*, *Streptococcus gordonii*, and other related species [3]. Some members are primary colonizers of the human oral cavity, and are considered relatively benign members of the oral microbial flora [1], [2], [4], [5], [6]. Nevertheless, members of this group can be responsible for a variety of infectious complications, including bacteremia and infective endocarditis [1], [6], [7], [8], [9]. The rate of bacteremia caused by the mitis group is reported to be similar to that caused by group A or group B streptococci [9]. Furthermore, epidemiological studies have shown the presence of these streptococcal species in heart valve and atherosclerotic plaque specimens [10], [11], [12].

Among the members of the mitis group of streptococci, *S. pneumoniae, S. mitis*, and *S.oralis* are closely related and exhibit >99% 16S rRNA sequence identity, making them difficult to distinguish using conventional biochemical tests [1], [3], [6], [13]. *S. pneumoniae* is a well-known human pathogen, and *S. mitis* occasionally causes a variety of infectious complications including infective endocarditis, bacteremia, and septicemia [1], [6], [9]. It is noted that the mitis group of streptococci produces hydrogen peroxide (H_2_O_2_) [1], [2], [6], which is considered to play important roles in bacterial competition in microbial communities such as oral biofilms [14], [15]. *S. sanguinis* and *S. gordonii*, other members of the oral mitis group, are reported to produce sufficient quantities of H_2_O_2_ to reduce the growth of many oral bacteria, including the cariogenic *Streptococcus mutans* and several periodontal pathogens [14], [15].

Recently, we found that *S. oralis* induces macrophage cell death *in vitro* due to H_2_O_2_-mediated cytotoxicity [16]. The cytotoxic effects of streptococcus-derived H_2_O_2_ on macrophages is also observed with *S. sanguinis* [16], [17], suggesting that H_2_O_2_ may contribute to the pathogenicity of the members of the oral mitis group of streptococci.

H_2_O_2_ is the simplest peroxide, and a strong oxidizer. H_2_O_2_ is also a known cytotoxic and tissue-damaging agent [18], [19]. Therefore, H_2_O_2_ produced by oral streptococci can disturb the host defense system in multiple ways. Since epithelial cells form the first line of host defense against many human pathogens [20], [21], we investigated the susceptibility of epithelial cells to infection by H_2_O_2_-producing oral streptococci.

## Materials and Methods

### Bacterial Strains and Culture Conditions

*S. oralis* ATCC 35037, a type strain originally isolated from the human mouth [22], was obtained from the Japan Collection of Microorganisms at the RIKEN Bioresource Center (Tsukuba, Japan). The pyruvate oxygenase gene (*spxB*)-deletion mutant (*spxB* KO) and the revertant mutant (*spxB* Rev), that possesses the wild-type allele, were generated from the wild type (WT) *S. oralis* ATCC 35037, as described previously [16]. The concentrations of H_2_O_2_ produced by the *S. oralis* WT and *spxB* Rev strains are estimated to be 1–2 mM, whereas that produced by *spx*B KO mutant are less than 0.2 mM [16].

*S. sanguinis* ATCC 10556, *S. mutans* MT8148 and *Streptococcus salivarius* HHT were selected from the stock culture collection in the Department of Oral and Molecular Microbiology, Osaka University Graduate School of Dentistry. They are representative strains of each streptococcal species, and widely used in the studies of the oral microbiology [1], [2], [3], [12], [16], [23], [24]. *S. mutans* and *S. salivarius* are not the members of the mitis group [1], [2], [3], and they do not produce H_2_O_2_ [1], [2]. These bacteria were cultured in Brain Heart Infusion (BHI) broth (Becton Dickinson, Sparks, MD, USA) at 37°C in a 5% CO_2_ atmosphere.

### Cell Culture

Human nasopharyngeal epithelial Detroit 562 cells (American Type Culture Collection, Manassas, VA, USA), bronchial epithelial Calu-3 cells (American Type Culture Collection), and cervical epithelial HeLa cells (RIKEN Bioresource Center) were cultured in Eagle’s minimum essential medium alpha (α-MEM; Invitrogen, Carlsbad, CA, USA) supplemented with 10% fetal bovine serum (FBS) (Invitrogen) (10% FBS α-MEM), penicillin (100 U/ml), and streptomycin (100 µg/ml) at 37°C in a 5% CO_2_ atmosphere.

### Epithelial Cell Death

Streptococcal strains were grown to the exponential phase and centrifuged at 5000×*g* for 5 min. Pelleted cells were then resuspended in 10% FBS α-MEM containing no antibiotics. Epithelial cells (2×10^5^ cells) in 24-well culture plates (Asahi Glass, Tokyo, Japan) were infected with viable streptococcal strains at a multiplicity of infection (MOI) of 50, 100, or 200, in the absence of antibiotics, for 2 h. Cells were washed with phosphate buffered saline (PBS, pH 7.2) to remove extracellular non-adherent bacteria, and cultured for 18 h in fresh medium containing antibiotics. Cells were then stained with 0.2% trypan blue (Sigma Aldrich, St. Louis, MO, USA) in PBS, and the numbers of viable and dead cells were counted using light microscopy (Nikon TMS-F, Nikon, Tokyo, Japan). One additional measure of cell death was whether the cells detached from the culture plates. The morphological changes of the infected cells were also determined using a phase-contrast microscope (Axiovert 40C, Carl Zeiss, Oberkochen, Germany). Cell death induced by H_2_O_2_ was determined using similar methods. Epithelial cells were treated with 1, 5, or 10 mM H_2_O_2_ (Nacalai Tesque, Kyoto, Japan) for 2 h, washed with PBS, and cultured for 18 h in fresh medium. The viability was determined by trypan blue staining.

### Effect of Catalase on Cell Viability

Prior to infection, 10 or 100 U/ml of catalase (Sigma-Aldrich) was added to the cultures of epithelial cells, and the cells were then infected with viable *S. oralis* WT (MOI; 50, 100, or 200) for 2 h. Cells were washed with PBS, and cultured in fresh medium containing catalase and antibiotics for 18 h. Viability was determined as described above.

### Enzyme-linked Immunosorbent Assays (ELISAs) for Interleukin-6 (IL-6) and β-defensin 2

Detroit 562 cells were infected with viable *S. oralis* WT, *spxB* KO, and *spxB* Rev strains (MOI; 50, 100 or 200) in the absence of antibiotics for 2 h. Other cultures were also treated with H_2_O_2_ (1, 5, and 10 mM) for 2 h. These cells were washed twice with PBS, and cultured in fresh medium containing antibiotics for an additional 18 h. Lipopolysaccharide (LPS) from *Escherichia coli* O111:B4 (1 µg/ml; Sigma-Aldrich) was used as a positive control. The concentrations of IL-6 and β-defensin 2 in the culture supernatants were measured using the IL-6 ELISA kit (R&D Systems, Minneapolis, MN, USA) and the β-defensin 2 ELISA kit (Phoenix Pharmaceuticals, Burlingame, CA, USA), respectively, according to the manufacturer’s instructions.

### Statistical Analysis

Statistical analyses were performed using QuickCalcs software (GraphPad Software, La Jolla, CA, USA). Experimental data are expressed as the mean ± SD of triplicate samples. Statistical differences were examined using independent Student’s *t*-test, with *p*<0.05 considered to indicate statistical significance.

## Results

### *S. oralis* Induces Epithelial Cell Death

We previously reported that infection with members of the oral mitis group of streptococci such as *S. oralis* and *S. sanguinis* induce THP-1 macrophage cell death, with bacterial H_2_O_2_ apparently contributing to this process [16], [17]. Our further study suggested that these streptococci are also capable of inducing cell death in other cell types, including epithelial cells.

**Figure 1:**
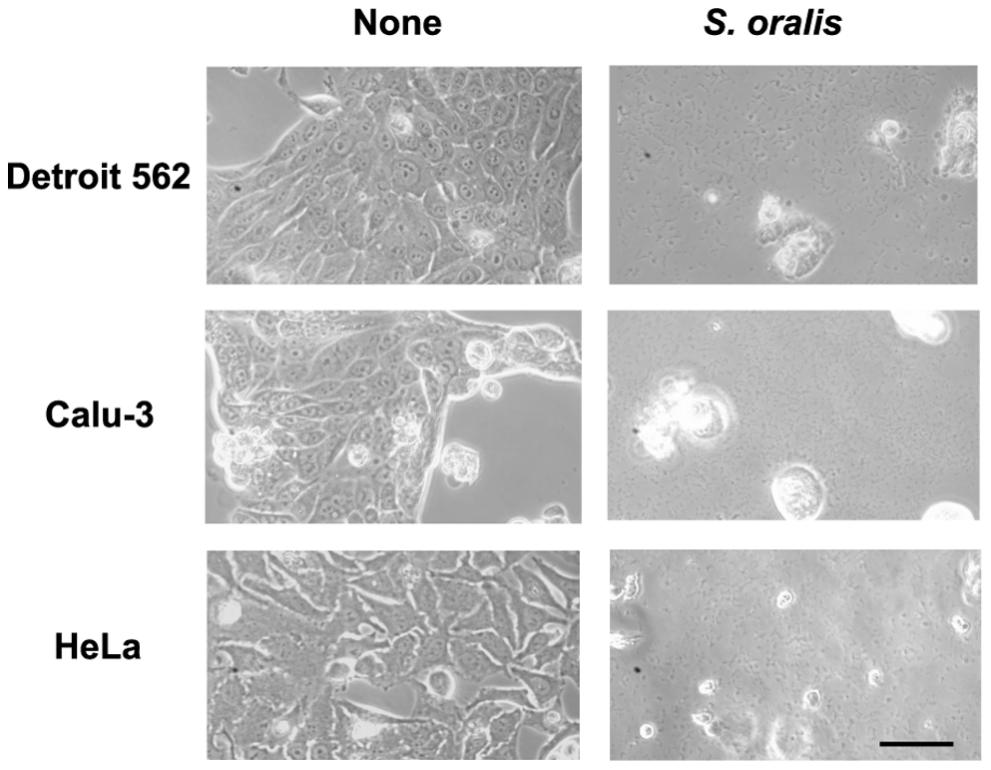
*S. oralis* induces epithelial cell death. Detroit 562, Calu-3, and HeLa epithelial cells (2×10^5^ cells) in 24 well culture plates were infected with viable *S. oralis* ATCC 35037 for 2 h, washed with PBS to remove non-adherent extracellular bacteria, and cultured in fresh medium containing antibiotics for 18 h. Changes in cellular morphology were observed using a phase-contrast microscope. Bar = 20 µm.

Epithelial cell lines Detroit 562, Calu-3, and HeLa were infected with viable *S. oralis* ATCC 35037 at an MOI of 200 for 2 h in antibiotics-free medium. Cells were washed with PBS to remove extracellular non-adherent bacteria. At this point, no change in cellular morphology was observed, and almost all cells appeared viable. However, after 18 h in culture in fresh medium containing antibiotics, epithelial cell death was apparent. Microscopic examination revealed that more than 80% of the epithelial cells were driven into cell death by *S. oralis* infection (Figure 1). Infected cells were detached from the bottom of the culture plates, and trypan blue staining confirmed the reduction of cell viability.

**Figure 2:**
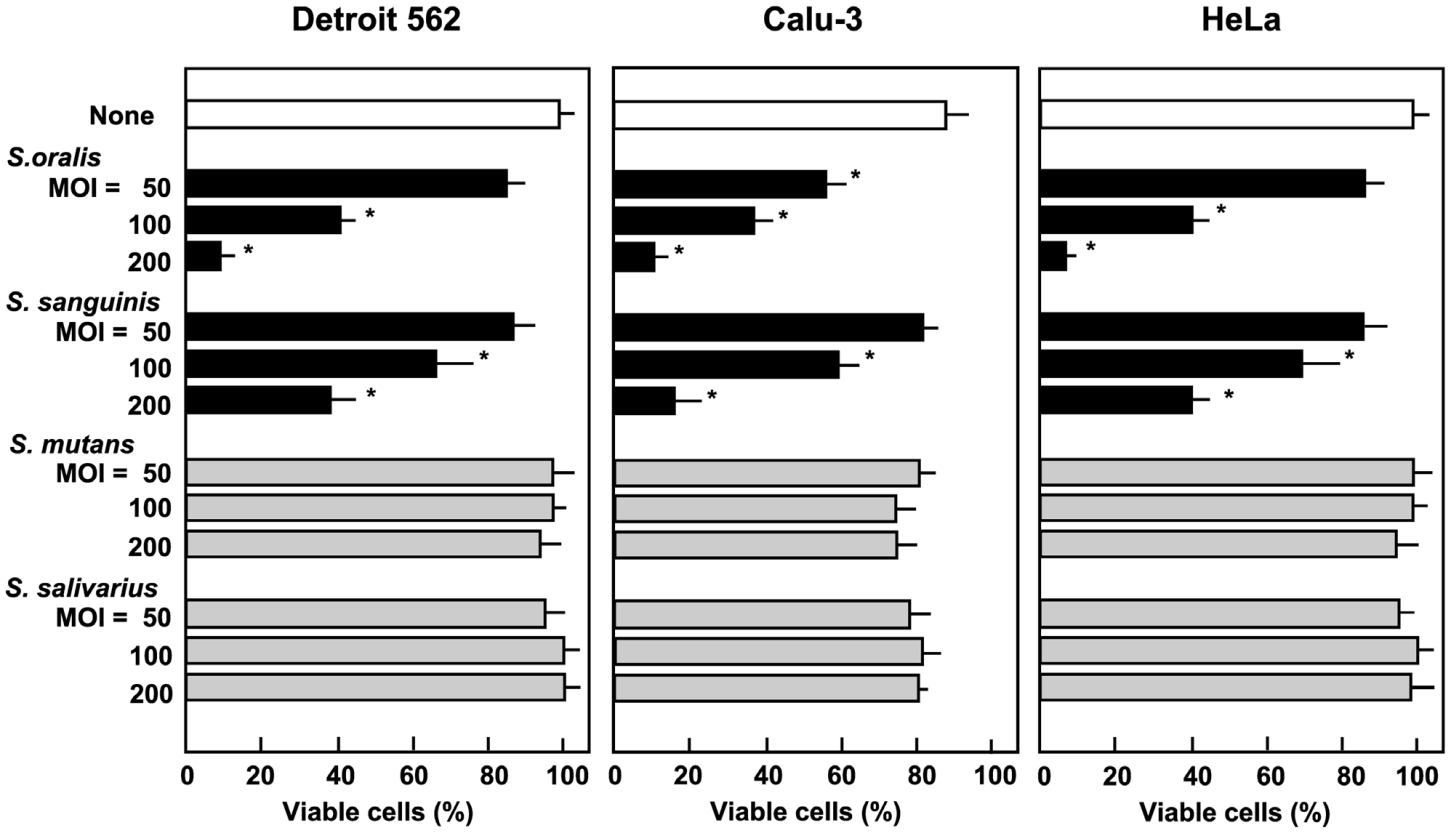
Epithelial cell death induced by oral streptococci. Detroit 562, Calu-3, and HeLa cells (2×10^5^ cells) in 24 well culture plates were infected with viable *S. oralis* ATCC 35037, *S. sanguinis* ATCC 10556, *S. mutans* MT8148, or *S. salivarius* HHT (MOI: 50, 100, or 200) for 2 h. The cells were then washed with PBS to remove non-adherent extracellular bacteria, and cultured in fresh medium containing antibiotics for 18 h. Viable cells were counted after trypan blue staining. Data are shown as the mean ± SD of triplicate samples. *p<0.05 as compared with untreated control (None).

We then examined whether other oral streptococcal species are capable of inducing epithelial cell death. Detroit 562, Calu-3, and HeLa cells were exposed to viable oral streptococcal strains, *S. oralis* ATCC 35037, *S. sanguinis* ATCC 10556, *S. mutans* MT8148, and *S. salivarius* HHT. After infection, cells were stained with trypan blue to determine cell viability (Figure 2). At an MOI of more than 100, *S. oralis* and *S. sanguinis* caused the cell death of epithelial cells. Exposure to *S. mutans* or *S. salivarius* had no effect on the viability of the cells even at an MOI of 200. These results suggest that the H_2_O_2_-producing oral mitis group may induce epithelial cell death. At an MOI of over 500, all tested streptococci steadily elicited cell death, but this was likely due to acidification of culture medium and/or accumulation of cytotoxic products such as formic and acetic acids (data not shown) [1], [2], [25].

### Streptococcal H_2_O_2_ Contributes to Epithelial Cell Death

**Figure 3:**
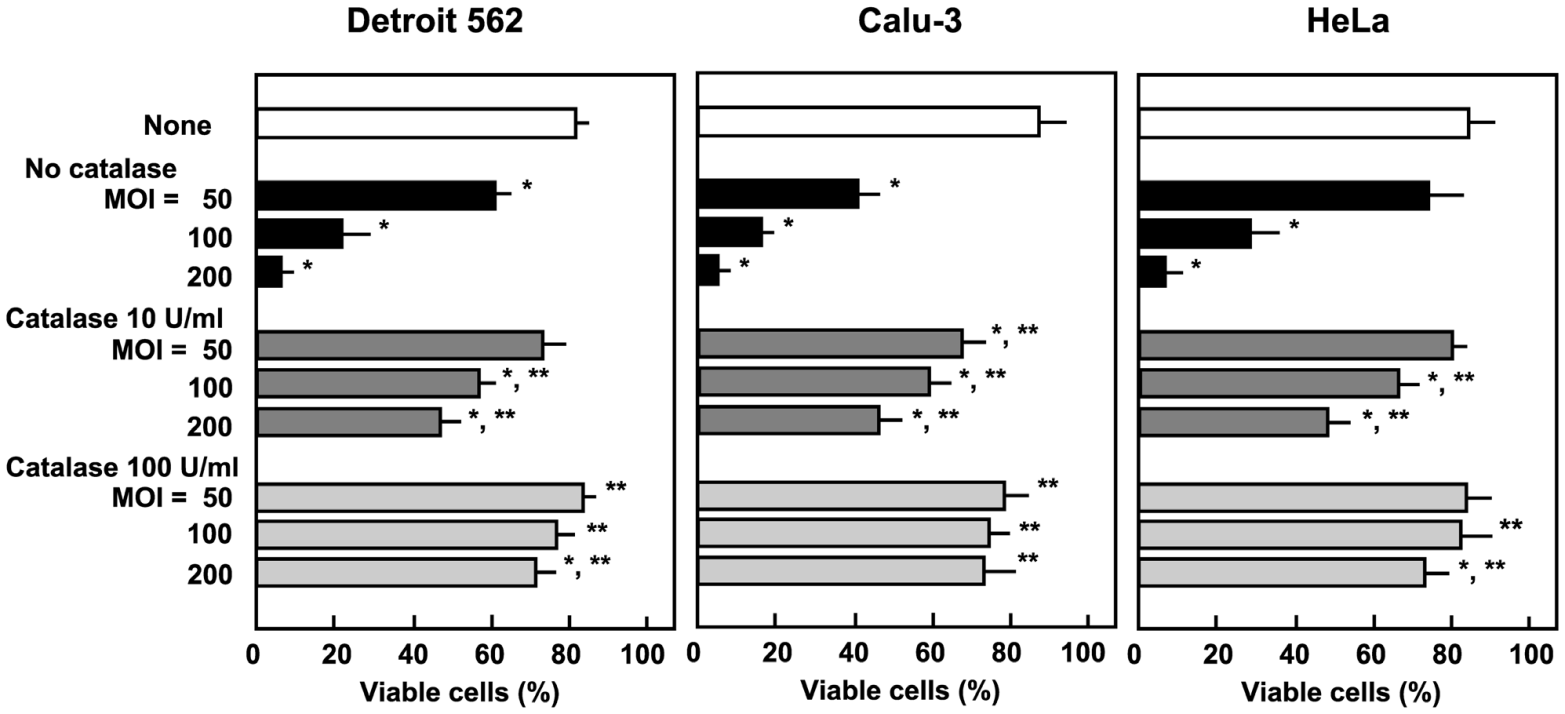
Effect of catalase on epithelial cell death. Prior to infection, either 10 or 100/ml of catalase was added to cultures of epithelial cells, and the cells were then infected with viable *S. oralis* ATCC 35037 (MOI: 50, 100, or 200) for 2 h. Cells were washed with PBS and cultured in fresh medium containing catalase and antibiotics for 18 h. Viability was determined using the trypan blue dye exclusion method. Data are shown as the mean ± SD of triplicate samples. *p<0.05 as compared with untreated control (None). **p<0.05 as compared with the cells infected at the same MOI without catalase.

In order to determine the contribution of H_2_O_2_ to *S. oralis*-induced cell death, the effect of catalase, an H_2_O_2_-decomposing enzyme, on cells infected with *S. oralis* was investigated. Exogenously added catalase was shown to reduce cell death in Detroit 562, Calu-3, and HeLa cells infected with *S. oralis* ATCC 35037 (Figure 3), suggesting that H_2_O_2_ is involved in the death of infected epithelial cells.

**Figure 4:**
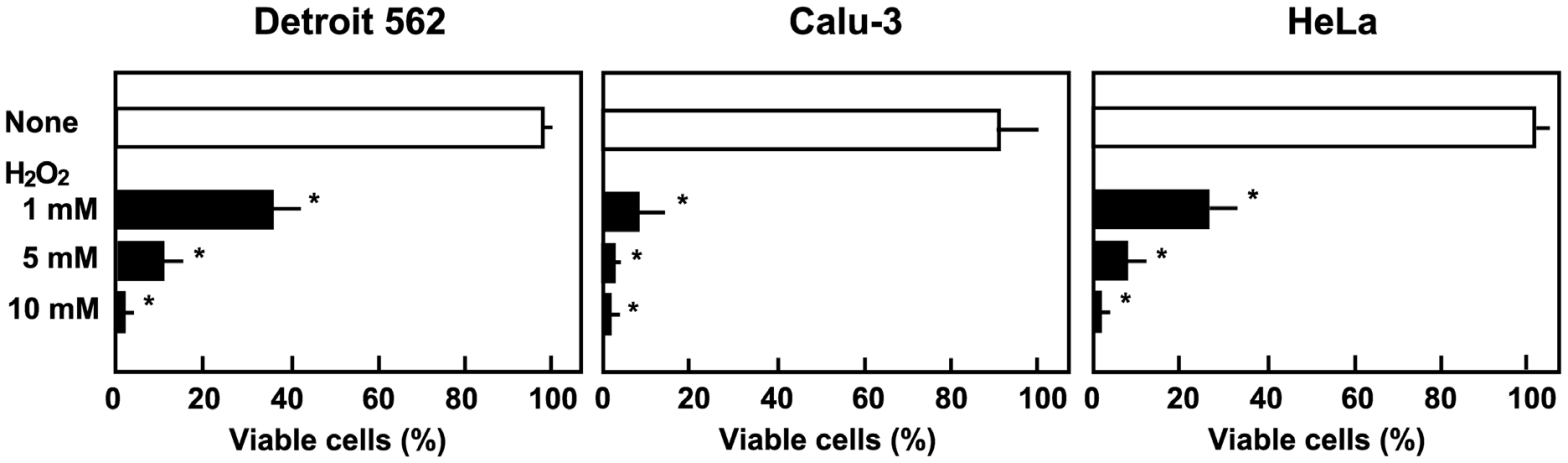
Cell death induced by H_2_O_2_. Detroit 562, Calu-3, and HeLa cells (2×10^5^ cells) in 24 well culture plates were cultured in the presence of 1, 5, or 10 mM H_2_O_2_ for 2 h, washed with PBS, and cultured in fresh medium for 18 h. Viability was determined using trypan blue staining. Data are shown as the mean ± SD of triplicate samples. *p<0.05.

To confirm that H_2_O_2_ alone is sufficient to induce cell death, these cell lines were incubated with H_2_O_2_. Epithelial cells were exposed to H_2_O_2_ at concentrations of 1, 5, or 10 mM for 2 h, and then washed with PBS to remove any remaining H_2_O_2_. Almost all cells were viable at this point. However, epithelial cell death was observed after 18 h in culture with fresh medium. Moreover, induced cell death occurred in a dose-dependent manner (Figure 4).

### Reduced Epithelial Cell Cytotoxicity of the *spxB* KO Mutant

**Figure 5:**
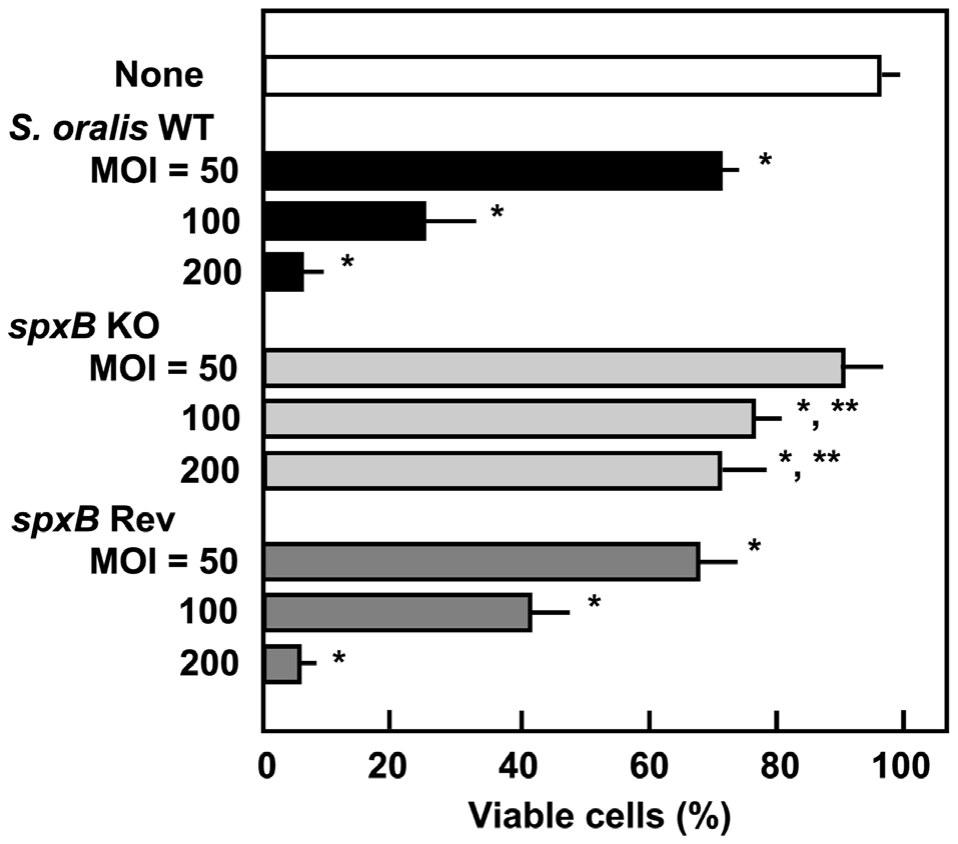
H_2_O_2_ contributes to the *S. oralis* cytotoxicity. Detroit 562 epithelial cells (2×10^5^ cells) in 24 well culture plates were infected with *S. oralis* WT, *spxB* KO, or *spxB* Rev strains (MOI: 50, 100, or 200) for 2 h, washed with PBS, and cultured in fresh medium containing antibiotics for 18 h. Viable cells were counted after trypan blue staining. Data are shown as the mean ± SD of triplicate samples. *p<0.05 as compared with untreated control (None). For the *spxB* KO, **p<0.05 as compared with the cells infected with WT at the same MOI.

Pyruvate oxidase has been reported to be a key enzyme for H_2_O_2_ production in the mitis group of streptococci [14], [16], [26], [27]. Therefore, we constructed a deletion mutant (*spxB* KO) of the pyruvate oxidase gene from *S. oralis* ATCC 35037 WT strain [16]. In order to elucidate the contribution of H_2_O_2_ produced by *S. oralis* to epithelial cell death, Detroit 562 cells were infected with *S. oralis* WT, *spxB* KO, or *spxB* Rev strains. *S. oralis* WT and *spxB* Rev strains induced Detroit 562 cell death in a dose-dependent manner (Figure 5). In contrast, *spxB* KO mutants showed reduced cytotoxicity, even at an MOI of 200, suggesting that streptococcal H_2_O_2_ plays a critical role in the observed cell death.

### Effect of *S. oralis* Infection and H_2_O_2_ on Inflammatory Mediator Production in Detroit 562 Cells

**Figure 6:**
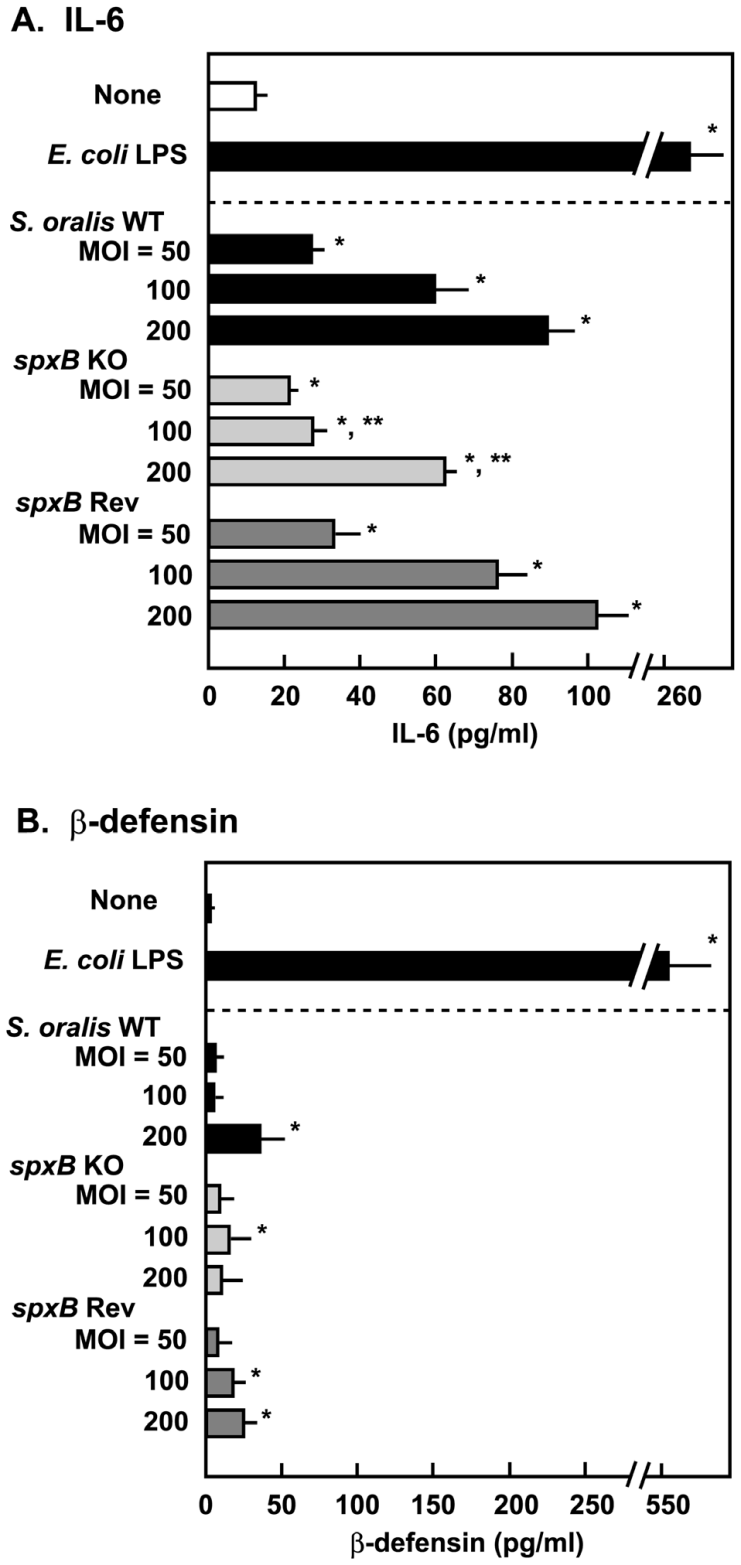
IL-6 and β-defensin 2 production by *S. oralis* strains. Detroit 562 epithelial cells (2×10^5^ cells) in 24 well culture plates were infected with viable *S. oralis* WT, *spxB* KO, or *spxB* Rev strains for 2 h, and then washed and cultured in fresh medium for 18 h. *E. coli* LPS (1 µg/ml) was used as a positive control. The release of IL-6 (A) and β-defensin 2 (B) was determined by ELISA. Data are shown as the mean ± SD of triplicate samples. *p<0.05 as compared with untreated control (None). For the *spxB* KO infected cells, **p<0.05 as compared with the cells infected with WT at the same MOI.

Bacterial infection is known to induce the proinflammatory response in a wide variety of host cells. In this study, we investigated whether *S. oralis* stimulates the production of IL-6 and β-defensin 2 by Detroit 562 epithelial cells. Detroit 562 cells were exposed to viable *S. oralis* strains or H_2_O_2_. As shown in Figure 6A, *S* *. oralis* infection enhanced IL-6 production by Detroit 562 cells, and the amount of IL-6 in the culture supernatants increased in a dose-dependent manner. IL-6 production by cells infected with *spxB* Rev mutant was comparable to that of the WT strain, while reduced IL-6 production was observed from cells infected with the *spxB* KO mutant. We also measured β-defensin 2 concentrations in culture supernatant. The quantities of defensin produced by cells infected with *S. oralis* were less than one-tenth of cells treated with *E. coli* LPS (Figure 6B), suggesting that the streptococcal infection induces less defensin production than *E. coli* LPS treatment does.

**Figure 7:**
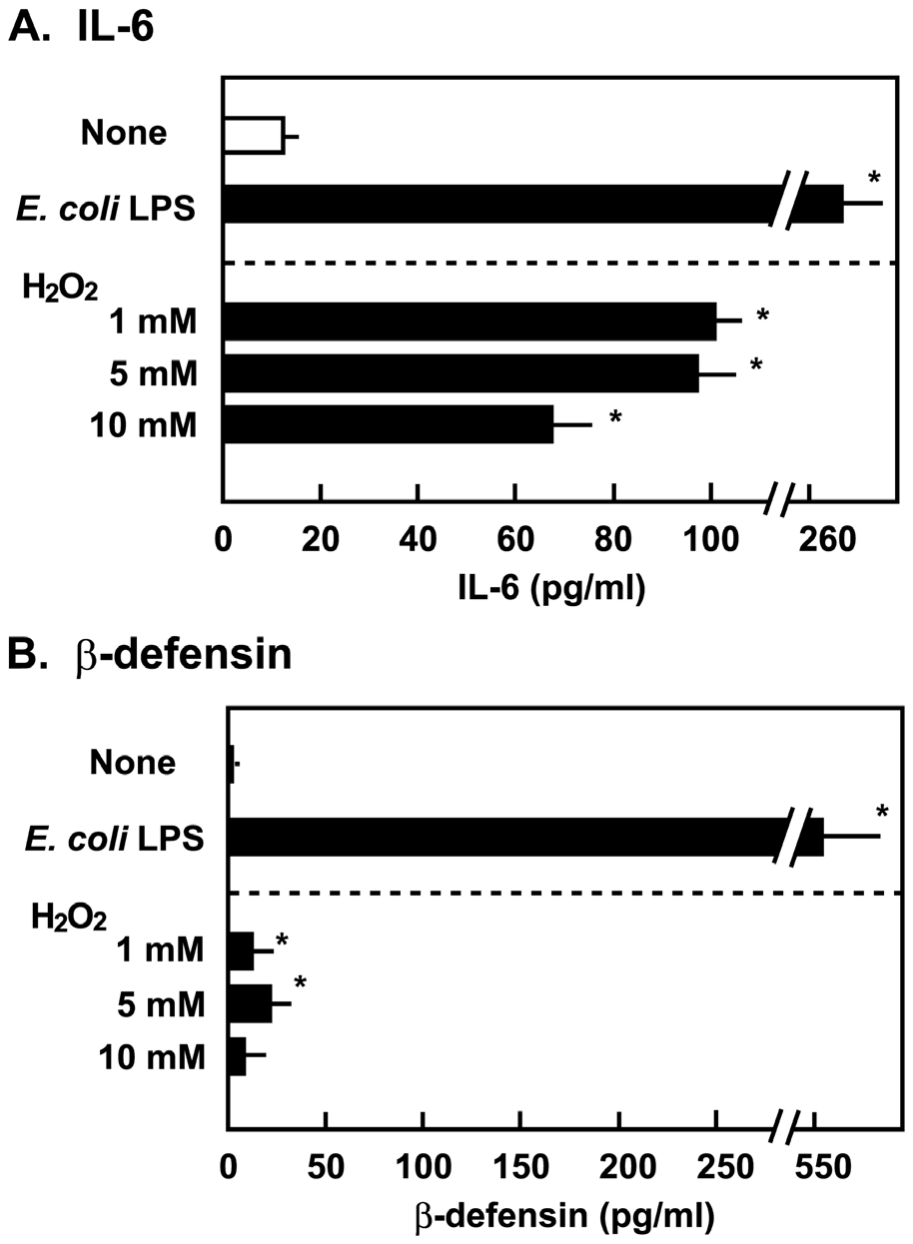
IL-6 and β-defensin 2 induction by H_2_O_2_. Detroit 562 epithelial cells (2×10^5^ cells) in 24 well culture plates were treated with H_2_O_2_ (1, 5, or 10 mM) for 2 h, and then washed and cultured in fresh medium for 18 h. *E. coli* LPS (1 µg/ml) was used as a positive control. The release of IL-6 (A) and β-defensin 2 (B) was determined by ELISA. The assays were performed simultaneously with those in Figure 6. Data for the negative (None) and positive (*E. coli* LPS) controls, which appeared in Figure 6, are duplicated. Data are shown as the mean ± SD of triplicate samples. *p<0.05 as compared with untreated control (None).

Further, we investigated the effect of H_2_O_2_ on IL-6 and β-defensin production. H_2_O_2_ (1 to 10 mM) itself stimulated IL-6 production in Detroit 562 cells (Figure 7A). Even at the cytotoxic levels, H_2_O_2_ were likely to be able to promote IL-6 production. On the other hand, the quantities of defensin produced by the cells treated with H_2_O_2_ were considerably less than that produced by cells treated with *E. coli* LPS (Figure 7B). Based on the induction of β-defensin 2 production, viable *S. oralis* and H_2_O_2_ appear to elicit a different response as compared with *E. coli* LPS.

## Discussion

The present study showed that *S. oralis* is capable of inducing epithelial cell death. The ability to induce cell death is presumably dependent on streptococcus-derived H_2_O_2_. H_2_O_2_ produced by *S. oralis* can also stimulate IL-6 production in epithelial cells, suggesting that this small oxidative molecule triggers some proinflammatory responses. Given our previous finding that H_2_O_2_ produced by *S. oralis* participates in macrophage cell death [16], these results suggest that various types of host cells are susceptible to the cytotoxic effect of H_2_O_2_. It should be noted that the epithelial cells were still viable after 2 h exposure to *S. oralis*. Therefore, cell death found in this study was not a simple acute reaction. The involvement of apoptosis [28] and/or pyroptosis [29] in the epithelial cell death was not investigated in the present study. However, the fact that the dead cells detached from the bottom of the culture plates suggests some contribution of anoikis, a detachment-induced cell death [30].

H_2_O_2_ is the simplest peroxide and a strong oxidizer, and is therefore considered a reactive oxygen species (ROS) [18], [19], [31]. The members of the oral mitis group of streptococci are reported to produce H_2_O_2_ at concentrations sufficient to kill other oral bacteria [14], [15]. In addition to the bactericidal activity, our present study revealed that H_2_O_2_ produced by *S. oralis* exhibits cytotoxicity to epithelial cells. The oral mitis group is known to cause a variety of infectious complications, including bacteremia and infective endocarditis [6], [7], [8], [9], [10], [11], [12]. The cytotoxicity and tissue-damaging effects of H_2_O_2_ may contribute to the pathogenicity of these bacteria. Therefore, it is likely that streptococcal H_2_O_2_ enables bacteria to escape from macrophage phagocytosis, and damages epithelial barriers, thereby contributing to bacterial dissemination.

Host defense against invading pathogens in the oral cavity and upper respiratory tracts relies mainly on the barrier function and innate immune system of epithelium [20], [21], [32], [33]. Bacterial pathogens penetrate the epithelium either through its disruption or by directly invading the epithelial cells [21]. In addition, exposure to H_2_O_2_ is reported to significantly increase the permeability of epithelial monolayers with a disruption of actin cytoskeleton [34], [35]. A similar disruption of actin cytoskeleton was observed in epithelial cells infected with *S. oralis* (data not shown), suggesting that streptococcal H_2_O_2_ can impair the integrity of the epithelial barrier.

H_2_O_2_ is also a virulence factor of *S. pneumoniae*, a pathogenic member of the mitis group [26], [36], [37]. In experimental animals, the oxidative molecule is suggested to exacerbate pneumococcal lung and blood infections [26] and nasopharyngeal colonization [37]. Another study showed that H_2_O_2_ produced by *S. pneumoniae* induces microglial and neuronal apoptosis *in vitro* [36]. These studies also demonstrate that H_2_O_2_ acts as a cytotoxin that contributes to the virulence of the mitis group of streptococci.

Originally, H_2_O_2_ was only considered to be lethally cytotoxic at high concentrations such as 0.9 M, which is the concentration of the commercially available 3% H_2_O_2_ solution [19], [20]. However, in the past few years, it has gained attention as a potential signaling molecule at subtoxic levels [38], [39], [40], [41]. H_2_O_2_ is now thought to influence signaling pathways that induces some proinflammatory responses [34], [38], [41]. In this study, we found that infection with viable *S. oralis* or exposure to H_2_O_2_ induced IL-6 production in Detroit 562 epithelial cells (Figures 6 and 7). IL-6 is a pleiotropic proinflammatory cytokine that acts on various cells [42], [43]. IL-6 promotes the differentiation of B cells and T cells, and thus amplifies immune and inflammatory responses. It also plays a critical role in autoimmune diseases such as rheumatoid arthritis [42], [43]. Several studies have demonstrated that subtoxic levels of H_2_O_2_ and other ROS stimulate the production of IL-6 in epithelial cells [34], [44], [45]. These results are in agreement with our findings in this study. Further, we measured another proinflammatory cytokine, interleukin-1β (IL-1β) in the culture supernatants, however, no significant IL-1β production could be detected even in the LPS-stimulated culture (data not shown).

Epithelial cells protect themselves from microbial pathogens through the production of antimicrobial peptides including defensins [20], [21], [32], [33]. β-defensins are small cationic peptides with broad-spectrum antimicrobial activity [32], [33]. In this study, we investigated the role of streptococcal H_2_O_2_ on β-defensin 2 production in Detroit 562 epithelial cells. The stimulatory effect of H_2_O_2_ on β-defensin production in epithelial cells was weak, whereas *E. coli* LPS, which was used as a positive control, strongly enhanced its production (Figure 7). Thus, H_2_O_2_ is thought to differentially regulate the expression of IL-6 and β-defensin 2 in epithelial cells. Several studies have reported that the oral mitis group of streptococci can stimulate cytokine and defensin productions in epithelial cells [46], [47], [48]. Ji et al. [46] reported that viable *S. sanguinis* enhanced IL-1α production in human gingival epithelial cells. They showed that *S. sanguinis* does not induce β-defensins and cathelicidin expression. On the other hand, Hasegawa et al. [47] described that viable *S. gordonii* inhibits IL-6 and interleukin-8 secretion from gingival epithelial cells. Therefore, infection with these oral streptococci seems to evoke multiple effects on epithelial cells. One potential reason for this difference could be the cytotoxicity of H_2_O_2_.

In conclusion, our study reveals that streptococcus-derived H_2_O_2_ is a potential cytotoxin. Furthermore, this simple oxidative molecule acts as an inducer of IL-6 production. These results strongly suggest that H_2_O_2_ contributes to the pathogenesis of the oral mitis group of streptococci.
